# An emerging trend of equal authorship credit in major public health journals

**DOI:** 10.1186/s40064-016-2771-7

**Published:** 2016-07-15

**Authors:** Shui-Ying Lei, Yan-Ping Dong, Wei-Fang Zhu, Lan-Juan Li

**Affiliations:** Editorial Office of Hepatobiliary and Pancreatic Diseases International, First Affiliated Hospital, College of Medicine, Zhejiang University, 79 Qingchun Road, Hangzhou, 310003 China; Department of Dermatology, First Affiliated Hospital, College of Medicine, Zhejiang University, 79 Qingchun Road, Hangzhou, 310003 China; The State Key Laboratory for Diagnosis and Treatment of Infectious Diseases, First Affiliated Hospital, College of Medicine, Zhejiang University, 79 Qingchun Road, Hangzhou, 310003 China; Collaborative Innovation Center for Diagnosis and Treatment of Infectious Diseases, 79 Qingchun Road, Hangzhou, 310003 China

**Keywords:** Public health, Authorship, Credit, Equal, Contributions

## Abstract

**Background:**

This study aimed to identify the longitudinal trends and characteristics of the practice of explicitly giving equal credit to multiple authors of publications in public health journals. Manual searches were conducted to identify original research articles, published in five public health journals with the highest IFs according to the “2012 JCR Science Edition” between January 1, 2004 and December 31, 2013, which awarded equal credit to multiple authors (*Epidemiologic Reviews*, *Environmental Health Perspectives*, the *International Journal of Epidemiology*, *Epidemiology*, and the *Annual Review of Public Health*). The Instructions to Authors in the five journals were also examined with regard to information about giving equal credit to multiple authors.

**Findings:**

Statistically significant differences were noted in the annual prevalence in *Environmental Health Perspectives*, *International Journal of Epidemiology*, and *Epidemiology* (*r* = 0.753, *P* = 0.012; *r* = 0.894, *P* = 0.000; *r* = 0.522, *P* = 0.122, respectively). The first two authors listed in the by-line received equal credit in the majority of articles, but this practice was also extended to authors in nearly every position on the by-line in some publications. The authors given equal credit in articles appearing in *Environmental Health Perspectives*, *International Journal of Epidemiology*, and *Epidemiology* were primarily from European and North American countries. Finally, none of the journals provided specific guidance regarding this practice in their Instructions to Authors.

**Conclusions:**

An emerging trend of giving equal credit to multiple authors is observed in the public health journals. This practice should be better addressed in the guidance provided by journals to authors.

## Background

Authors who have contributed substantially to original research should be listed in the by-line (International Committee of Medical Journal Editors 2016). Nowadays research is mostly collaborative involving many workers with different contributions. Thus multiple authorship becomes common in publications. During the past decade, international scientific collaboration has strongly intensified in not only Europe but also across the world (Arunachalam and Doss 2000). Leaders of different research groups that participate in these international/national scientific collaborations may play almost the same role in their individual groups. Simply listing authors in the by-line cannot reflect properly each author’s contribution in these international/national scientific collaborations. Therefore, articles with authors given equal credit emerged (Akhabue and Lautenbach 2010; Wang et al. 2012; Tao et al. 2011).

Since 2000, several biomedical journals began to publish articles with multiple authors given equal credit. A study (Akhabue and Lautenbach 2010) investigated this phenomenon in five general medical journals (the *New England Journal of Medicine*, the *Journal of the American Medical Association*, the *Lancet*, the *Annals ofInternal Medicine*, and the *British Medical Journal*) from 2000 to 2009 and noted an increasing trend toward according multiple authors with equal credit as a function of time. Specifically, the first two authors listed in the by-line received equal credit in the majority of articles, but the practice was also applied to authors in nearly every position in the by-line on some occasions. However, only the *Lancet*’s Instructions to Authors explicitly note that all the authors in the by-line can be credited with having made an equal contribution. Another study (Wang et al. 2012) found a similar increasing trend in four critical care medicine journals published from 2001 to 2010 and also noted that majority of the authors given equal credit were primarily from European and North American countries. One study (Tao et al. 2011) found that the authors of articles appearing in four major anaesthesiology journals who were given equal credit were from different countries and regions and had a variety of funding sources.

It has been reported that biomedical journals account for the majority of publications in which multiple authors are given equal credit (Wu et al. 2009). However, research regarding this practice in public health journals has not been conducted. This study explored the tendency of public health journals to give multiple authors equal credit and examined differences among disciplines in this regard.

## Methods

We used the applications “Journal Citation Report” (JCR) and “Web of Science” to analyse relevant data in the Institute for Scientific Information (ISI) Web of Knowledge database. We selected the five public health-related journals under the subject category of “Public, environmental and occupational health” with the highest IFs according to the “2012 JCR Science Edition”: *Epidemiologic Reviews*, *Environmental Health Perspectives*, the *International Journal of Epidemiology*, *Epidemiology*, and the *Annual Review of Public Health*.

We limited our search of the Web of Science database to “articles” and “reviews” that were published in the aforementioned journals between 2004 and 2013. Letters to the Editor, book reviews, editorials, and other kinds of published material were excluded. We then downloaded the search results, including all available abstracts. Two reviewers then manually searched the full text of the articles in the publisher databases of *Epidemiologic Reviews*, the *International Journal of Epidemiology*, *Epidemiology*, and the *Annual Review of Public Health* as well as those on the website of *Environmental Health Perspectives*, which is an open-access journal. The footnotes as well as the descriptions of the author contributions at the end of the main text were examined for information regarding equal authorship. The Instructions to the Authors of each journal were also reviewed on December 24, 2014 to determine if guidance regarding the assignment of equal credit was offered.

The total number of articles and reviews and the number of articles in each journal in which multiple authors were given equal credit were calculated for each year in the study period. We also recorded the total number of authors, the number of authors given equal credit and their position in the by-line (i.e. first author, middle author, and last author), the year of publication, and the affiliations of all authors (country, institution, and department) for each article. We then calculated the annual proportion of articles in which multiple authors were given equal credit treating the total number of original research articles published in each journal per year as the denominator. The position of authors given equal credit, the median total number of authors, the proportion of authors given equal credit in articles in which multiple authors were given equal credit, and the regional distribution of authors were also recorded.

### Statistical analysis

Statistical analyses were performed using the SAS 9.0 software (SAS Institute, Cary, NC). The Microsoft Excel 2003 software was used to establish the database. Bivariate-related research relied on linear correlations or Spearman rank correlations to analyse the growth-related trends in journals publishing articles from 2004 to 2013 in which multiple authors were given equal credit. A *P* value of <0.05 was considered to indicate statistical significance.

## Results

During the study period of 2004 and 2013, totally 5509 papers were published in the five public health journals. Among them, a total of 152 articles giving equal credit to multiple authors were identified in four of the five journals (0 in the *Annual Review of Public Health*; 1 in *Epidemiologic Reviews*, 6 in *Epidemiology*, 98 in *Environmental Health Perspectives*, and 47 in the *International Journal of Epidemiology*). Of the 98 articles giving equal credit to multiple authors published in the *Environmental Health Perspectives*, two articles appeared in 2004, whereas 12 appeared in 2013 (<1 vs. 5.8 %). The *International Journal of Epidemiology* published a total of 47 articles giving equal credit to multiple authors, one of these appeared in 2004, whereas 11 appeared in 2013 (<1 vs. 8.3 %). A trend toward an increased number of articles giving equal credit to multiple authors was observed in *Environmental Health Perspectives* and the *International Journal of Epidemiology*, with the most prominent increase in 2009 (Table 1). However, trends toward annual increases in the number of articles giving equal credit to multiple authors were also noted in *Environmental Health Perspectives*, the *International Journal of Epidemiology*, and *Epidemiology* (*r* = 0.753, *P* = 0.012; *r* = 0.894, *P* = 0.000; *r* = 0.522, *P* = 0.122, respectively). The IFs of the five selected public health journals increased steadily during the study period, and no direct relationship between equality of authorship and IF was observed. Of the five public health journals, *Epidemiologic Reviews* with the highest IF of 7.583 published only one article of this sort; while the *Annual Review of Public Health* with the lowest IF of 5.451 did not publish any articles giving equal credit to multiple authors. We excluded the *Annual Review of Public Health* from further analyses since it does not publish articles giving equal credit to multiple authors in the study period.

**Table 1:** Number of articles with equal credit given to multiple authors in the five public health journals in 2004–2013

Year	*Epidemiologic Reviews*	*Environmental Health Perspectives*	*International Journal of Epidemiology*	*Epidemiology*	*Annual Review of Public Health*
2004	0/10 (0 %)	2/252 (<1 %)	1/130 (<1 %)	0/89 (0 %)	0/24 (0 %)
2005	0/10 (0 %)	4/310 (1.3 %)	1/139 (<1 %)	0/105 (0 %)	0/24 (0 %)
2006	0/11 (0 %)	4/343 (1.2 %)	1/145 (<1 %)	0/89 (0 %)	0/22 (0 %)
2007	0/12 (0 %)	6/304 (2.0 %)	2/128 (1.6 %)	0/94 (0 %)	0/22 (0 %)
2008	0/10 (0 %)	13/272 (4.8 %)	0/133 (0 %)	0/95 (0 %)	0/25 (0 %)
2009	1/11 (9.1 %)	15/303 (5.0 %)	6/141 (4.3 %)	0/109 (0 %)	0/21 (0 %)
2010	0/13 (0 %)	17/270 (6.3 %)	7/161 (4.3 %)	0/110 (0 %)	0/30 (0 %)
2011	0/13 (0 %)	15/288 (5.2 %)	6/121 (5.0 %)	0/92 (0 %)	0/26 (0 %)
2012	0/14 (0 %)	10/268 (3.7 %)	12/128 (9.4 %)	0/88 (0 %)	0/25 (0 %)
2013	0/13 (0 %)	12/208 (5.8 %)	11/133 (8.3 %)	6/101 (5.9 %)	0/24 (0 %)
Total	1/117 (<1 %)	98/2818 (3.5 %)	47/1359 (3.5 %)	6/972 (<1 %)	0/243 (0 %)
*r*, *P* value	*r* = 0.058, *P* = 0.873	*r* = 0.753, *P* = 0.012	*r* = 0.894, *P* = 0.000	*r* = 0.522, *P* = 0.122	–

The numerator represents the number of articles with equal credit given to multiple authors: the denominator represents the total number of articles published in a certain year in that journal

Most of the articles in *Environmental Health Perspectives*, the *International Journal of Epidemiology*, and *Epidemiology* that gave equal credit to multiple authors during the study period gave such credit to the first two authors (63 articles, 64.3 %; 32, 68.1 %; and 3, 50.0 %, respectively). This practice was applied to the last two authors with the second most frequency (13, 13.3 %; 5, 10.6 %; and 2, 33.3 %, respectively), but it was also applied to authors in nearly every position in the by-line on some occasions (Table 2). Both authors of the article in *Epidemiologic Reviews* were given equal credit.

**Table 2:** Number of articles with equal credit given to multiple authors according to by-line position in 2004–2013

By-line position of authors given equal credit	*Epidemiologic Reviews* (*n* = 1)	*Environmental Health Perspectives* (*n* = 98)	*International Journal of Epidemiology* (*n* = 47)	*Epidemiology* (*n* = 6)	Total (*n* = 152)
First two authors	0	63 (64.3 %)	32 (68.1 %)	3 (50.0 %)	98 (64.5 %)
First three or more authors	0	5 (5.1 %)	3 (6.4 %)	1 (16.7 %)	9 (5.9 %)
Last two authors	0	13 (13.3 %)	5 (10.6 %)	2 (33.3 %)	20 (13.2 %)
First and last author^a^	1 (100 %)	4 (4.1 %)	4 (8.5 %)	0	9 (5.9 %)
First two and last two authors	0	3 (3.1 %)	1 (2.1 %)	0	4 (2.6 %)
Middle authors only	0	2 (2.0 %)	1 (2.1 %)	0	3 (2.0 %)
First three authors or more					
Last three authors or more	0	1 (1.0 %)	0	0	1 (0.7 %)
All authors^b^	0	3 (3.1 %)	0	0	3 (2.0 %)
Others	0	4 (4.1 %)	1 (2.1 %)	0	5 (3.3 %)

^a^For articles with only two authors, we considered the authors as first author and last author

^b^Excluded articles with only two authors

The median total number of authors of articles in *Environmental Health Perspectives*, the *International Journal of Epidemiology*, and *Epidemiology* that gave equal credit to multiple authors in 2004–2013 were 8.5 (2–24), 9 (2–65), and 11.5 (6–14), respectively; the median total number of equally credited authors was two (2–9), two (2–5), and two (2–3), respectively (Table 3). In general, two authors were given equal credit in those articles that awarded equal credit to multiple authors appearing in *Environmental Health Perspectives*, the *International Journal of Epidemiology*, and *Epidemiology* (84 articles, 85.7 %; 42, 89.4 %; and 5, 83.3 %, respectively). An article in *Environmental Health Perspectives* gave the most authors (nine) equal credit.

**Table 3:** Median total number of authors and number of authors of articles in which multiple authors are given equal credit in 2004–2013

Number of authors	*Epidemiologic Reviews*	*Environmental Health Perspectives*	*International Journal of Epidemiology*	*Epidemiology*
Number of authors listed in by-line (median, range)	2 (2)	8.5 (2–24)	9 (2–65)	11.5 (6–14)
Number of equally credited authors (median, range)	2 (2)	2 (2–9)	2 (2–5)	2 (2–3)

Articles in *Environmental Health Perspectives*, the *International Journal of Epidemiology*, and *Epidemiology* in which equal credit was given to multiple authors were written primarily by scholars from Europe, North America, and Asia (based on the region of the corresponding author in articles that gave equal credit). Scholars from Europe, North America, and Asia given equal credit in articles accounted for 35.7, 40.8, and 23.5 %, respectively, in *Environmental Health Perspectives*; these figures were 55.3, 14.9, and 23.4 %, respectively, for the *International Journal of Epidemiology*; and 33.3, 16.7, 50 %, respectively, for *Epidemiology* (Table 4). Additional analysis revealed that the authors of 39.8 % (39/98) of the articles in *Environmental Health Perspectives* that gave equal credit to multiple authors came from the US; this was followed by China (16, 16.3 %), France (6, 6.1 %), England (6, 6.1 %), and Germany (5, 5.1 %). Articles in the *International Journal of Epidemiology* that gave equal credit to multiple authors were written primarily by scholars from the UK (13, 27.7 %), followed by China (7, 14.9 %), the US (6, 12.2 %), France (3, 12.5 %), and Switzerland (3, 12.5 %). The authors of articles in *Epidemiology* giving equal credit to multiple authors were primarily from China (3, 50 %), followed by the US, France, and Norway (1 each, accounting for 16.7 %). Both authors of the article in *Epidemiologic Reviews* that gave equal credit to multiple authors were from the US.

**Table 4:** Authors of articles with equal credit given to multiple authors by region in 2004–2013

Region	*Epidemiologic Reviews* (*n* = 1)	*Environmental Health Perspectives* (*n* = 98)	*International Journal of Epidemiology* (*n* = 47)	*Epidemiology* (*n* = 6)	Total (*n* = 152)
Europe	0	35 (35.7 %)	26 (55.3 %)	2 (33.3 %)	63 (41.4 %)
North America	1 (100 %)	40 (40.8 %)	7 (14.9 %)	1 (16.7 %)	49 (32.2 %)
Asia	0	23 (23.5 %)	11 (23.4 %)	3 (50.0 %)	37 (24.3 %)
Africa	0	0	1 (2.1 %)	0	1 (<1 %)
Austria	0	0	2 (4.3 %)	0	2 (1.3 %)

More than half (57.0 %) of the authors of articles in *Epidemiologic Reviews*, *Environmental Health Perspectives*, the *International Journal of Epidemiology*, and *Epidemiology* that gave equal credit to multiple authors were from different affiliations, whereas, 43 % of these cases were from the same affiliation (Table 5). The percentage of the authors given equal credit from different affiliations were 58.2 (57/98), 51.1 (24/47), and 66.7 % (4/6), respectively in *Environmental Health Perspectives*, the *International Journal of Epidemiology*, and *Epidemiology*. In addition, none of these public health journals mentions the requirements for equal contribution in its Instructions to Authors.

**Table 5:** Affiliations of authors of articles with equal credit given to multiple authors in 2004–2013

Affiliations	*Epidemiologic Reviews* (*n* = 1)	*Environmental Health Perspectives* (*n* = 98)	*International Journal of Epidemiology* (*n* = 47)	*Epidemiology* (*n* = 6)	Total (*n* = 152)
Different affiliations	1 (100 %)	57 (58.2 %)	24 (51.1 %)	4 (66.7 %)	86 (56.6 %)
Same affiliation^a^	0	41 (41.8 %)	23 (48.9 %)	2 (33.3 %)	66 (43.0 %)

^a^Authors with overlapping affiliations were considered to have the same affiliation

## Discussion

Similar to the previous reports (Wang et al. 2012; Akhabue and Lautenbach 2010; Tao et al. 2011), we found that the authors that were most frequently given equal credit were the first two, followed by the last two; however, authors in every position of the by-line were given equal credit in some articles. The most common number of authors to receive equal credit was two, and more than half of the articles in which multiple authors were given equal credit were written by scholars from different affiliations. Although we observed an increasing trend toward major public health journals publishing more articles giving equal credit to multiple authors, this is not so common in the *Annual Review of Public Health* and *Epidemiologic Reviews*, which only published 0 and 1 such article, respectively in the study period.

We did not find a direct relationship between the number of articles giving equal credit to multiple authors and the IF of the journal. The five public health journals under examination (2012 JCR report) were established in the 1970s or 1980s, and their IFs have increased steadily over the past 10 years. A study by Conte et al. also reported an increase of co-first authorship in biomedical and clinical publications in both the high- (*Cell*, *Science*, *Nature*, *JAMA*, *Lancet*, and the *New England Journal of Medicine*) and mid- (*FASEB Journal*, *Journal of Cell Science*, *Oncogene*, *American Journal of Gastroenterology*, the *Archives of Internal Medicine*, and *Heart*) IF journals (Conte et al. 2013). Articles giving equal credit to multiple authors accounted for 33.3 % of the total papers published in *Science*, which is considerably higher than the comparable figures for journals from Mainland China during the same period (Wu et al. 2009). Thus, the tendency to publish articles giving equal credit to multiple authors is not related to the category of the journal, the number of the articles it publishes annually, or its IF.

In recent years, the number of authors per article has been increasing. Research regarding a series of journal conducted by the Danish Medical Association in 2011 found that the total number of authors of all types of articles has increased; in 1960, articles with more than three authors accounted for 1 % of all articles, but this figure increased to 68 % in 2010. The median number of authors of original articles in 1960, 1985, and 2010 were two (1–3), three (1–9), and three (1–9), respectively. Review articles accounted for one (1–2), two (1–5), and three (1–14), respectively, whereas case reports accounted for one (1–2), two (1–5), and three (1–6), respectively (Vinther and Rosenberg 2012). Some scholars have pointed out that some articles with multiple authors accorded equal credit to some portion thereof (Schreiber 2009). This increase in the number of authors may reflect the close cooperation involved in international scientific research projects.

In this study, the majority of articles in which equal credit was given to multiple authors were written by scholars from European and North American countries, the majority from the UK and US. More than half (57.0 %) of the authors of articles in *Epidemiologic Reviews*, *Environmental Health Perspectives*, the *International Journal of Epidemiology*, and *Epidemiology* that gave equal credit to multiple authors were from different affiliations. This finding is consistent with results from Tao et al.(2011), who reported that the majority of the authors gave equal credit in four anaesthesiology journals came from different countries or regions and articles were supported by various funding sources. This situation is attributable to multidisciplinary or multicentre collaboration.

Because the first and last authors receive the most credit for an article, the other co-authors do not receive much attention for their involvement (Lei and Li 2012). In 1997, Smith called for recognition of the time contributed to authorship (Smith 1997). In the 2000s, journals began to include a specific section that specified each author’s contribution (Lei et al. 2012; Qian 2002). Of the five selected public health journals, only *Epidemiologic Reviews* includes Instructions to Authors that mandate that the requirements of authorship are specified and reproduces the International Committee of Medical Journal Editors’ (ICMJE) specification about authorship. *Epidemiology* stipulates that authors meet the ICMJE’s requirements of authorship. None of these journals mentions the requirements for equal contribution in its Instructions to Authors (Epidemiologic reviews; Annual Review of Public Health; EHP; Epidemiology; International Journal of Epidemiology). *JAMA*, *Nature*, and the *Lancet* require a statement about each author’s contribution and provide a description of giving equal credit to authors, but no specific requirement regarding how to define equal credit is included (*JAMA*; *Nature*; *Lancet*). Although no guidelines about giving equal credit were available in the early 2000s, an increasing number of journals adopted the following description after 2010: “These authors contributed equally to this work”.

The analysis of the ScienceDirect database from 1989 to 2008 showed a tendency toward “smooth–slow growth–significant growth” in giving equal credit to multiple authors each year, this was especially evident in the 2000s (Wu et al. 2009). This phenomenon was observed in pharmacy as well as in biological medicine (Dotson 2013). The awarding of equal credit to a number of authors seems to be a common practice. We also noted that the authors of 43 % of the articles with equal credit given to multiple authors were from the same affiliations. As giving equal credit to authors may have implications for promotion decisions, questions of academic misconduct may arise in such situations. Furthermore, the inclusion of multiple first authors as well as a corresponding author may dilute the responsibility of the corresponding author. Scientific journals focus on reporting research results, and the authors take responsibility for issues related to authorship and the division of credit. International ethics bodies, such as the World Association of Medical Editors (WAME), the ICMJE, and the Committee on Publication Ethics (COPE) (WAME; ICMJE; COPE) have issued clear rules about authorship but not about equal credit. In this context, academic promotion committees should apply scientifically and logically valid evaluation criteria.

## Limitations

The present study has one potential limitation. We focused primarily on the five public health journals with the highest IFs. Although we examined current data regarding articles with equal credit given to multiple authors, our results may not be representative of other journals.

## Conclusions

In conclusion, although not all of the five journals studied published articles in which equal credit was given to multiple authors, it is increasingly common in public health journals to publish articles given equal credit to multiple authors. Thus, we recommend that journals should include specific requirements regarding giving authors equal credit in the section outlining the Instructions for Authors. Academic promotion committees should strengthen the standards used to manage this practice, and a scientifically and logically valid evaluation system should be developed to promote the further refinement of this practice. International institutions, such as the ICMJE, World Association of Medical Editors (WAME), and COPE should provide relevant guidelines for use by scholars and journal editors.
